# Involvement of HIV patients in treatment-related decisions

**DOI:** 10.7448/IAS.17.4.19600

**Published:** 2014-11-02

**Authors:** Matteo Zanini, Kathrin Schulte-Hermann, Birgit Leichsenring, Wiltrut Stefanek, Thomas Ernst Dorner

**Affiliations:** 1 Specialty, MSD Austria, Vienna, Austria; 2 Medical Department, MSD Austria, Vienna, Austria; 3 Medical Information/Dokumentation, AIDS Hilfe Wien, Vienna, Austria; 4 PULSHIV, Patient Organisation, Vienna, Austria; 5 Institute of Social Medicine, Centre for Public Health, Medical University of Vienna, Vienna, Austria

## Abstract

**Introduction:**

The improvement of antiretroviral therapy in the past decades has had a major impact on life expectancy and quality of life of people living with HIV, and also on the relationship between patients and their physicians. What used to be an acute treatment for life threatening complications, and an end-of-life therapy in the beginning of the epidemic, turned over the time into a lifelong care. The good relationship between patients and physicians represents the cornerstone of an optimal long-term therapy. Shared decision making between patients and physicians is a crucial prerequisite for the success of this approach. Several Austrian patient organizations developed an online survey together with MSD (the so-called “PAB-test”) aimed to evaluate how people living with HIV perceive the level of care in Austria.

**Materials and Methods:**

An online survey has been developed to evaluate how people living with HIV feel about the relationship with their physicians and to what extent they feel involved in treatment related decisions.

**Results:**

A total of 303 subjects completed the questionnaire. 44% felt “totally” involved in their therapy, 40% “strongly involved”, 12% “fairly involved”, 3% “poorly involved” and 1% “not at all involved” in their therapy. The proportion of subjects who felt totally involved in the therapy was equally distributed between sex, sexual orientation, age groups, groups of various education level, and between patients treated predominantly in intramural or extramural medical care. The most important factor for people living with HIV to feel involved in their therapy is a low amount of long-term ART-related side-effects (Figure 1).

**Conclusions:**

The results show that the majority of people living with HIV in Austria feel involved in therapy related decisions. This proportion is equally distributed in patients with different socio-demographic or socio-economic characteristics or level of medical care delivery. According to the survey, the most important reason for people living with HIV to be involved in their therapy is to avoid long-term side effects.

**Figure 1:**
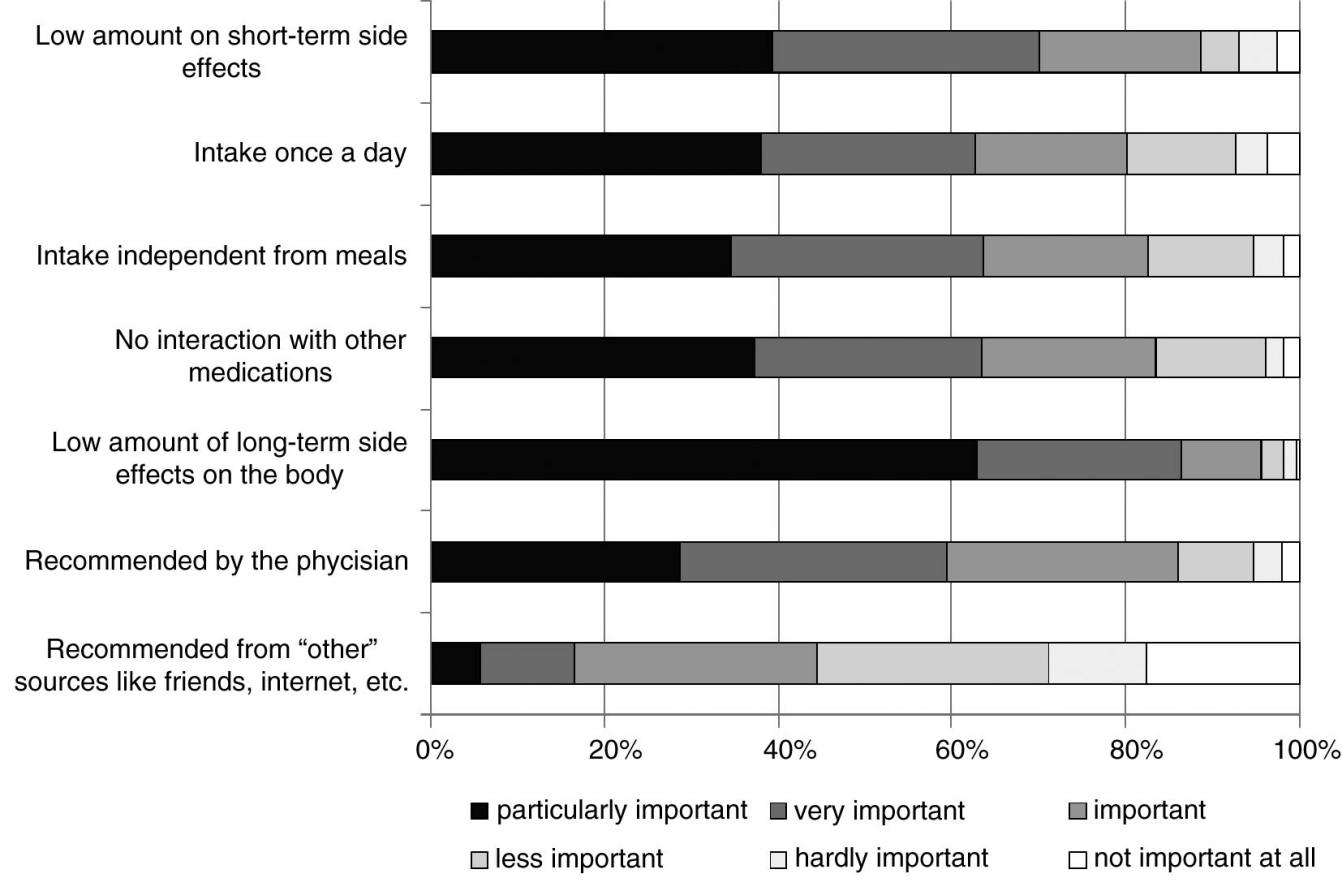
Importance of therapy-related factors for choice of ART.

